# QingXia HuaYu Formula Alleviates Severe Acute Pancreatitis–Associated Intestinal Injury by Modulating Group 3 Innate Lymphoid Cells

**DOI:** 10.1155/grp/6013880

**Published:** 2025-11-28

**Authors:** Yuyang Liu, Yishuang Tang, Yue Wu, Yujie Jiang, Liang Zhang, Mingxian Zheng, Yixuan Ye, Jing Kong, Xiaosu Wang, Lin Yuan, Feng He, Bingduo Zhou

**Affiliations:** ^1^Department of Gastroenterology, Yueyang Hospital of Integrated Traditional Chinese and Western Medicine, Shanghai University of Traditional Chinese Medicine, Shanghai, China; ^2^The Center for Cancer Research, School of Integrative Medicine, Shanghai University of Traditional Chinese Medicine, Shanghai, China; ^3^Department of Traditional Chinese Medicine, Jinhua Municipal Central Hospital, Zhejiang, China; ^4^Center for Science and Technology Experiments, Shanghai University of Traditional Chinese Medicine, Shanghai, China

**Keywords:** Group 3 innate lymphoid cells, intestinal injury, QingXia HuaYu, severe acute pancreatitis

## Abstract

**Background:**

Severe acute pancreatitis (SAP), one of the common causes of acute abdomen in clinical practice, is characterized by acute and rapid onset as well as the systemic inflammatory response syndrome, which often leads to intestinal injury and high mortality. Recent studies indicate that innate lymphoid cells (ILCs) play an important role in the connection of SAP and intestinal injury. TCM, including the QingXia HuaYu (QXHY) formula, is known to benefit gastrointestinal dysfunction in SAP, but its mechanisms, especially regarding ILCs, are unclear.

**Methods:**

A mouse model of SAP was established by retrograde infusion of sodium taurocholate into the common bile duct of C57BL/6 mice. QXHY was orally administered three times postinfusion. Serum inflammatory markers, pancreatic myeloperoxidase, and histopathological changes in the mice with SAP with or without QXHY treatment were used to evaluate the pancreatic injury and the therapeutic effects of QXHY. Intestinal tight junctions, serum markers for mucosal injury, and inflammatory factors were used to analyze the effects of QXHY on intestinal injury in SAP mice. Flow cytometry and qRT-PCR were used to analyze ILC3s numbers and functions.

**Results:**

QXHY significantly reduced serum amylase, pancreatic myeloperoxidase, and improved histopathological manifestations in SAP mice. In addition, QXHY reduced intestinal damage, improved tight junction integrity, and lowered proinflammatory cytokines. Mechanistically, QXHY restored the diminished ROR*γ*t^+^-ILC3s in SAP mice and upregulated the expression of IL-22 (interleukin-22) and IL-17 (interleukin-17) mRNA.

**Conclusion:**

QXHY mitigates SAP-associated intestinal damage by enhancing ILC3 cells to regulate tissue injury and maintain homeostasis. Our data suggest that QXHY is a promising alternative and cost-friendly therapy for SAP-associated intestinal damage.

## 1. Introduction

Acute pancreatitis (AP) is a common gastrointestinal disorder with a rising global incidence. Due to its unpredictable clinical course and significant mortality risk, AP often requires urgent medical intervention. Although most cases are self-limiting, approximately 10%–20% of patients develop severe acute pancreatitis (SAP), which is characterized by persistent organ failure and carries a high risk of death [1]. AP is a common gastrointestinal disorder with a rising global incidence. Due to its unpredictable clinical course and significant mortality risk, AP often requires urgent medical intervention. Although most cases are self-limiting, approximately 10%–20% of patients develop SAP, which is characterized by persistent organ failure and carries a high risk of death [2]. The lungs are the most frequently and severely affected organ; however, other organs such as the heart, kidneys, liver, and intestines may also be involved, contributing to the high mortality observed in SAP [3].

Gallstones, alcohol, and hypertriglyceridemia are primary etiological factors of AP. After AP onset, proteases, released due to pancreas damage, become preactivated and trigger autolysis of pancreatic acinar cells and disruption of vascular endothelial cells, which further leads to augmentation of tissue injury and inflammation [2]. Early occurrence of intestinal injury is also observed, and it can further result in the onset of multiple organ failure (MOF) [4]. This increasing recognition underscores the interdependence of intestinal compromise and concomitant organ complications in AP. A collection of both clinical and experimental research confirms the vulnerability of intestinal immune defenses, perturbation of intestinal microbiota equilibrium, augmented apoptosis of intestinal epithelial cells (IECs), and reduced expression of tight junctions (TJs), concomitant with increased proinflammatory cytokine levels [5–10]. The resultant shifts in the intestinal barrier integrity in AP promote the translocation of bacteria and endotoxins (ETs) into the bloodstream. In this milieu, an increase of proinflammatory mediators is activated, exacerbating both local and systemic AP severity [11, 12].

Currently, no specific medicine exists for treating pancreatitis. Standard AP treatment emphasizes symptom and complication management through medical interventions targeting inflammatory pathways, along with optimized supportive care, including fluid resuscitation, pain management, and nutritional support [13]. The preservation of intestinal homeostasis has emerged as a critical imperative in the pursuit of effective AP management. In recent years, clinicians have investigated various strategies for modulating intestinal microecology and its associated roles. Among these strategies, early enteral nutrition (EN) is one of the most broadly adopted treatments for AP. This approach sustains intestinal functions and acts as a preventive measure against secondary infectious complications [14]. Studies have shown that early EN reduces in-hospital mortality in patients with SAP and mitigates the risk of MOF to some extent [15]. Nevertheless, the prevalence of early EN intolerance in SAP patients remains significantly high, reaching up to 58.7% [16]. Consequently, the clinical use of early EN in SAP cases is substantially limited.

Furthermore, various therapeutic interventions, including probiotic or prebiotic supplementation, antibiotic regimens, fecal microbiota transplantation (FMT), and medicinal interventions, have significantly improved the overall outcomes of AP treatment. However, variability in individual responses is important, along with the presence of adverse events and the high costs of certain medications [17, 18]. Western medical approaches also have shortcomings in addressing challenges including paralytic ileus, ET absorption, and bacterial translocation (BT). This emphasizes the desperate need for novel therapeutic avenues for AP that harmonize cost-effectiveness with robust gastrointestinal protective roles.

With a history of millennia, phytochemicals and traditional medicine, from which many phytochemicals are derived, have gradually gained popularity worldwide, especially in Japan, China, and India [19]. Traditional Chinese medicine (TCM) has been progressively embraced in China. It provides substantial benefits in managing AP, specifically in addressing its associated gastrointestinal dysfunction. Several individual TCM therapies have been extensively applied in AP patients. For instance, rhubarb, mirabilite, and herbal formulations including Da-Cheng-Qi decoction, Chaiqin Chengqi decoction, and Da Chaihu decoction, have garnered significant attention within the realm of AP treatment. Despite the widespread utilization, the lack of comprehensive molecular evidence has complicated the therapeutic efficacy of TCM.

QingXia HuaYu (QXHY) formula, a decoction rooted in TCM principles and developed by our clinical and research team, has demonstrated therapeutic effects on AP and AP-associated symptoms in both clinical trials and animal studies. As evidenced by its protective effects on the gastrointestinal tract and immune function [20], QXHY has been shown to improve intestinal barrier function in rats with AP, mediated through the p38MAPK/ATF2 signaling pathway [21]. Furthermore, studies have explored the influence of QXHY and its decomposed formulations on the intestinal mucosal barrier in experimental rat models of SAP [22]. More importantly, when combined with conventional Western medicine treatments, such as fasting, fluid replacement, anti-infection therapy, maintenance of acid–base and electrolyte balance, administration of enzyme inhibitors, and pancreatic secretion suppressants, QXHY exhibited superior beneficial effects compared to conventional Western treatment alone [23]. QXHY is composed of several herbal constituents, including red vine, *Bupleurum*, *Corydalis*, *Salvia*, red peony, Jiaoshan Gardenia, and raw rhubarb. Based on TCM theory, this formula is attributed with effects such as promoting defecation, clearing heat, regulating qi dynamics, and enhancing blood circulation [24]. Clinical studies showed that QXHY facilitates the recovery of gastrointestinal function in AP patients and shortens hospital stays [25]. In addition, animal studies incorporating histomorphological and serological analyses suggest that QXHY alleviates intestinal mucosal barrier impairment in rats with SAP. These therapeutic benefits position QXHY as a promising alternative treatment for SAP. However, the active ingredients of QXHY and their mechanisms of action remain to be fully elucidated.

Intestinal immune cells intricately mediate the integrity of the intestinal mucosal barrier, which is an essential component of both structural and functional intestinal aspects. Among these immune constituents, innate lymphoid cells (ILCs) have gradually received quite extensive attention in the last decade due to their capacity to resemble adaptive immune cells. This family of immune cells, which was recently discovered in the mucosal systems of both mice and humans, is critical in maintaining tissue homeostasis, regulating metabolism and tissue repair [26]. ILCs act as key regulators of gut homeostasis and provide promising avenues for novel interventions targeting intestinal damage. The most common ILC subtype within the intestinal mucosa is Group 3 innate lymphoid cells (ILC3s). ILC3s play a critical regulatory role in maintaining intestinal equilibrium by secreting cytokines interleukin-22 (IL-22) and IL-17 [27]. However, their role in the SAP is not yet studied.

This work investigated the therapeutic effect of QXHY on SAP-induced intestinal damage and revealed the mechanism of abundance and functionality of ILC3s. Using SAP mouse models, we showed that QXHY mitigated intestinal damage and exerted a constructive effect on the ILC3 population in the ileum. Our findings illuminate previously undisclosed facets of the potential of QXHY in countering SAP-associated intestinal impairment, thereby establishing a reference for future research and the ultimate clinical use of QXHY against SAP.

## 2. Methods

### 2.1. SAP Mouse Model

Male C57BL/6 mice of 6 weeks of age were purchased from Shanghai SLAC Laboratory Animal Co. Ltd. (Permission No. SCXK (hu) 2002-0006) and were acclimatized at the animal facility for 1 week before SAP modeling using the retrograde biliopancreatic duct injection method. Mice were fed with a standard chow diet and housed in specific pathogen-free (SPF) conditions according to standard laboratory protocols. The Institutional Animal Care and Use Committee of Shanghai University of TCM Affiliated Yueyang Hospital approved all animal experiments (YYLAC-2020-087).

### 2.2. Experimental Design and Animal Treatments

Mice were randomly allocated into three experimental groups: the sham surgery + vehicle treatment group (Sham + Veh), the SAP model + vehicle treatment group (SAP + Veh), and the SAP + QXHY treatment group (SAP + QXHY). To induce the SAP model, a freshly prepared solution of 2% sodium taurocholate (STC) (Sigma, CAS:145-42-6) was administered into a bile-pancreatic duct using a standard pressure-controlled infusion method (50 *μ*L/animal). This procedure was performed under the effect of 3% sodium pentobarbital anesthesia according to the established protocol [28]. In the Sham + Veh group, an equal volume of sterile saline instead of 2% STC was introduced using the same technique, followed by oral gavage of Veh. As QXHY was extracted with purified water, purified water was used as Veh for oral gavage administration in this study. The SAP + QXHY group received intragastric administration of QXHY solution (granule concentration 0.32 g/mL) at a dose of 15 mL/kg. Administration of QXHY or vehicle control was performed 0.5 h before STC or sham surgery and at 5 and 11 h postsurgery. Twenty-four hours after SAP modeling, samples were collected from animals following anesthesia with 2% sodium pentobarbital (50 *μ*L/animal).

### 2.3. Histopathology

Pancreatic and terminal ileum tissue sections were prepared and stained as previously described. Briefly, the tissues were meticulously dissected into 0.5 cm-thick segments, followed by desiccation, paraffin embedding, and fixation using 4% paraformaldehyde. Subsequently, conventional methodologies were used to create 5 mm paraffin cross-sections, for hematoxylin and eosin (HE) staining. We used Mayer's hematoxylin solution and 1% eosin solution from Wako for this purpose. The resulting histological sections were observed and captured under a Leica DMI4000B light microscope coupled with a Leica DFC310 FX digital camera for accurate depiction and documentation of histological features.

### 2.4. Transmission Electron Microscopy (TEM)

The ultrastructure of the intestinal TJs was assessed through TEM as described [29]. In brief, terminal ileum tissues were extracted from the mice and gently irrigated with 0.9% sterile saline to remove luminal contents. Subsequently, these tissues were sectioned into 1 mm cubes and immersed in 2% glutaraldehyde at a temperature of 4°C for more than 2 h. Thereafter, they underwent postfixation with 1% osmium tetroxide for 1 h, washed twice using phosphate-buffered saline (PBS), and then subjected to stepwise dehydration using ascending concentrations of ethanol. The specimens were subsequently exposed to a solution of Epon 812 and epoxypropane infiltrating, culminating in embedding in Epon 812 resin. To promote ultrathin sectioning, slices of 4 *μ*m thickness were excised from the samples and desiccated. Uranyl acetate staining (Ted Pella Inc., CA, United States) and lead citrate staining (Beijing Zhongjingkeyi Technology Company, Beijing, China) were used on the ultrathin sections. Selected regions of interest were then imaged using a TEM (FEI Tecnai Spirit; Hillsboro, Oregon, United States) for 10 min under ambient temperature conditions. After rinsing and drying, the ultrastructure of the TJs within the terminal ileum was observed using a JEM-1400 plus TEM (JEOL, Japan).

### 2.5. Enzyme-Linked Immunosorbent Assay (ELISA)

Quantification of myeloperoxidase (MPO) within pancreatic tissues (#CK-E20262), amylase (AMY) (#CK-E94521), interleukin-6 (IL-6) (#CK-E20012), interleukin-1*β* (IL-1*β*) (#CK-E20533), tumor necrosis factor-alpha (TNF-*α*) (#CK-E20262), D-lactate (D-LA) (#CK-E92970), diamine oxidase (DAO) (#CK-E20139), and ET (#CK-E20316) concentrations within the serum was executed using commercial ELISA kits from R&D Systems, Minneapolis, MN, United States. These assays were conducted following the manufacturer's prescribed guidelines and instructions.

### 2.6. qRT-PCR

Real-time quantitative polymerase chain reaction (RT-qPCR) was used to quantitate the expression of genes in tissues as previously described [30, 31]. Briefly, total RNA extraction from the terminal ileum tissues was conducted using the TRIzol reagent based on the established manufacturer's guidelines. The extracted RNA was subsequently subjected to reverse transcription, generating complementary DNA (cDNA), using the first-strand cDNA synthesis kit sourced from Allmeek Co. Ltd., Beijing, China. The quantification of relative mRNA levels of the designated target genes was performed using RT-qPCR. This molecular analysis was performed using a SYBR QPCR mixture, also from Allmeek Co. Ltd., Beijing, China, and was executed within the confines of the ABI 7500 real-time fluorescence quantitative PCR instrument.

Table 1 outlines the primer sequences integral to this analysis. The thermal cycling conditions were as follows: an initial predenaturation step at 95°C for 10 min, succeeded by a sequence of 40 cycles encompassing denaturation at 95°C for 10 s and annealing/extension at 60°C for 30 s. The quantified expressions of the target genes were subjected to normalization against the reference gene, *β*-actin, thereby affording a context of relative expression levels. The subsequent determination of fold changes was accomplished using the established 2^−ΔΔCT^ methodology.

### 2.7. Western Blot

Equivalent quantities of terminal ileum tissues were subjected to homogenization in RIPA lysis buffer (Beyotime, Shanghai, China, Cat. No. P0013E). After homogenization, centrifugation was performed at 12,000 rpm for 5 min, yielding the supernatant as the source of total protein content. The quantification of protein concentrations was conducted using a bicinchoninic acid (BCA) protein assay kit (Beyotime, Shanghai, China, Cat. No. WB2123).

The ensuing protein samples were subjected to separation using sodium dodecyl sulfate-polyacrylamide gel electrophoresis (SDS-PAGE) gels and subsequently transferred onto polyvinylidene difluoride (PVDF) membranes. After the transfer, the membranes were individually exposed to primary antibodies, including Anti-Claudin-1 (ab211737), Anti-Occludin (ab216327), Anti-ZO-1 (ab221546), and GAPDH (ab263962), during an overnight incubation at 4°C. Before antibody treatment, the membranes were preincubated with 5% BSA for 2 h at ambient room temperature. After antibody treatment, the membranes underwent a 2-h incubation with secondary antibodies conjugated with horseradish peroxidase (HRP). This secondary antibody treatment was preceded by a wash step using TBST buffer for 5 min, in triplicate. Ultimately, protein signals were observed using an ECL protein detection kit.

### 2.8. Flow Cytometric Analysis and Cell Sorting

Isolated single-cell suspensions (1 × 10^7^ cells/sample) subject to antibody staining under cold conditions (4°C) after the blockade of Fc receptors via the application of the 2.4G2 mAb (BD Pharmingen, Cat. No. 554656). Staining for deceased cells was conducted using LIVE/DEAD Fixable Aqua Dead Cell Stain (BD Pharmingen, Cat. No. 562247). This was followed by staining with the ensuing fluorochrome-labeled monoclonal antibodies: APC-CY7-anti-CD45 (BD Pharmingen, Cat. No. 557659), BV510-anti-CD4 (BioLegend, Cat. No. 563106), and FITC-conjugated lineage cocktail (CD3e, B220, Gr-1, CD11b, Ter-119; BioLegend, Cat. No. 133302). Identification of lymphocytes was contingent upon the expression of CD45, whereas ILCs were distinguished as CD45 + Lin-.

For the staining of transcription factors, after surface antigen staining as previously outlined, lymphocytes underwent fixation and permeabilization utilizing the Foxp3/Transcription Factor Buffer Set (Thermo Fisher Scientific, Cat. No. 00-5523-00) following the manufacturer's stipulations. Staining for T-bet (BV786, BioLegend, Cat. No. 564141), GATA3 (BB700, BioLegend, Cat. No. 566642), and ROR*γ*t (BV650, BioLegend, Cat. No. 564722) followed this process. ILC1, ILC2, and ILC3 subtypes were differentiated as CD45 + Lin-T-BET+, CD45 + Lin-GATA3+, and CD45 + Lin-ROR*γ*t+, respectively.

To assess intracellular cytokine production, cells were incubated for 3 h at 37°C in the presence of GolgiPlug (BD Biosciences). Subsequently, staining for IL-22 (APC, eBioscience, Cat. No. 17-7222-82) and IL-17A (PE, BioLegend, Cat. No. 560820) was executed after fixing with 4% PFA. The flow cytometric analysis of lymphocytes encompassed their initial classification via rigorous forward scatter (FSC) and side scatter (SSC) criteria, followed by gating grounded in CD45 expression. The process was conducted using a BD Celesta flow cytometer. Data analysis was performed using FlowJo (V10.0) software (BD, NJ, United States).

### 2.9. Statistical Analysis

Data were presented in the form of means ± standard deviation (SD). Statistical analysis and data visualization were conducted using SPSS 24.0 and GraphPad Prism 9.0 software. For comparisons among multiple groups, a one-way analysis of variance (ANOVA) was executed, followed by subsequent Tukey's post hoc testing. Significance levels established at *p* < 0.05 indicated significant differences (⁣^∗^*p* < 0.05, ⁣^∗∗^*p* < 0.01, and ⁣^∗∗∗^*p* < 0.001).

## 3. Results

### 3.1. QXHY Ameliorates Pancreatic Injury and Inflammation

**Figure 1:**
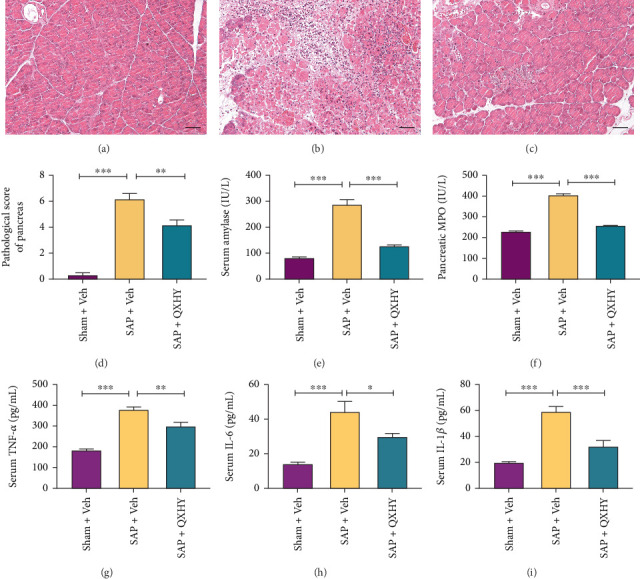
Protective effects of QHXY on pancreatic tissue injury and inflammation in SAP mice. (a–c) Representative images of HE-stained pancreas sections from mice in the indicated groups (×200). (d) Histopathological scores of pancreatic tissues from the indicated groups. (e) Serum AMY levels from the indicated groups. (f) MPO activity in pancreatic tissues for the groups shown. (g–i) Serum levels of inflammatory factors TNF-*α*, IL-6, and IL-1*β* in the indicated groups. Data are presented as mean ± SD (*n* = 6 per group) from at least three independent experiments: Sham + Veh, sham surgery + Veh group (Sham + Veh); SAP + Veh, the SAP model + vehicle treatment group (SAP + Veh); and QXHY, the SAP + QXHY treatment group (SAP + QXHY). Statistical significance levels are represented as ⁣^∗^*p* < 0.05, ⁣^∗∗^*p* < 0.01, and ⁣^∗∗∗^*p* < 0.001.

The initial assessment revolved around determining the efficacy of QXHY against STC-induced pancreatic tissue injury. Morphological analysis through HE staining showed a normative structural configuration of pancreatic tissue within the Sham + Veh group (shown in Figure 1a). Conversely, pancreatic tissue extracted from mice in the SAP group demonstrated noticeable manifestations of edema, hemorrhage, inflammatory cell infiltration, cellular necrosis, and compromised acinar as well as lobular architecture (shown in Figure 1b). Strikingly, within SAP mice treated with QXHY, significant reductions were noted in edema, hemorrhage, inflammatory cell infiltration, cellular necrosis, as well as the disarray of acinar and lobular structures, juxtaposed to their SAP counterparts (shown in Figure 1c). The subsequent pathological scoring of pancreatic tissues 24 h posttreatment was aligned with these observations (shown in Figure 1d).

In line with these findings, comparative analyses of serum AMY levels and MPO expression in pancreatic tissues showed significant reductions in the QXHY-treated SAP mice unlike those in the SAP group (shown in Figure 1e,f). Notably, serum samples taken 24 h posttreatment revealed increased levels of proinflammatory cytokines including TNF-*α*, IL-6, and IL-1*β* in the SAP animals, relative to the Sham + Veh mice. Intriguingly, the increased cytokine milieu was mitigated in SAP mice treated with QXHY (shown in Figures 1g, 1h, and 1i).

### 3.2. QXHY Alleviates SAP-Associated Intestinal Damage

**Figure 2:**
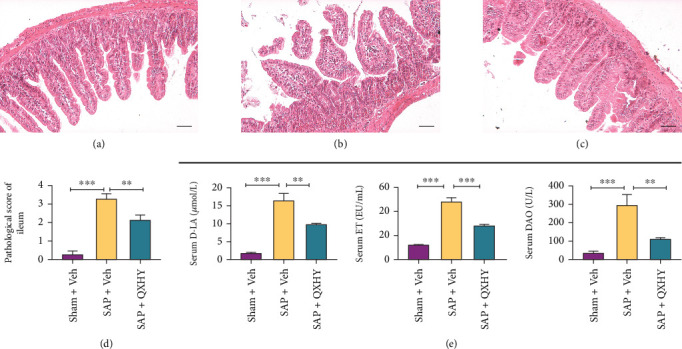
QHXY alleviates the ileal tissue injuries in SAP mice. (a–c) HE staining of ileal sections from the indicated mice groups (HE, ×200). (d) Histopathological scores allocated to ileal tissues for the indicated groups. (e) Quantification of serum levels of DAO, D-LA, and ET as markers of intestinal mucosal impairment in the indicated groups. The data are presented as mean ± SD values for each group (*n* = 6 per group) from at least three independent experiments: Sham + Veh, sham surgery + Veh group (Sham + Veh); SAP + Veh, the SAP model + vehicle treatment group (SAP + Veh); and QXHY, the SAP + QXHY treatment group (SAP + QXHY). Statistical significance levels are represented as ⁣^∗∗^*p* < 0.01, ⁣^∗∗∗^*p* < 0.001).

Subsequently, we investigated the potential of QXHY in counteracting SAP-induced intestinal impairment. Upon microscopic evaluation, the intestinal mucosa in the Sham + Veh group displayed a structurally normal appearance (shown in Figure 2a). However, within the model group, we noted notable signs of mucosal congestion, edema, shortened and detached villi, and compromised integrity, alongside the infiltration of inflammatory cells (shown in Figure 2b). Although edematous villi were present in the QXHY group, other pathological features were significantly mitigated (shown in Figure 2c). A consistently diminished pattern in pathological scores was evident within the QXHY group (shown in Figure 2d), unlike in the SAP group.

Moreover, serum concentrations of D-LA, DAO, and ET in the SAP group exceeded those in the control group (shown in Figure 2e). Comparatively, QXHY-treated SAP mice had reduced levels of these indicators (shown in Figure 2e), highlighting the efficacy of QXHY in ameliorating intestinal disturbances in SAP mice.

### 3.3. QXHY Preserves the Integrity of the Ileac Epithelial Barrier

**Figure 3:**
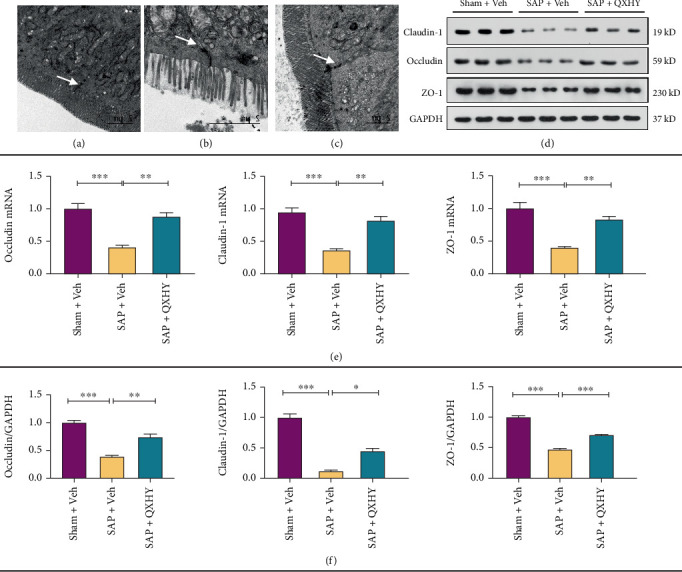
QXHY rescues the intestinal barrier and increases the expression of ileal epithelial tight junctions in SAP mice. (a–c) TEM images show the ileal tissue ultrastructure in the indicated groups (TEM, ×5000). (d, e) Detection of Claudin-1, Occludin, and ZO-1 mRNA levels in ileal tissues by qRT-PCR analysis. (f) Protein levels of Claudin-1, Occludin, and ZO-1 in ileal tissues determined by Western blotting. Data are presented as mean ± SD for each group (*n* = 3 per group). Sham + Veh, sham surgery + Veh group (Sham + Veh); SAP + Veh, the SAP model + vehicle treatment group (SAP + Veh); and QXHY, the SAP + QXHY treatment group (SAP + QXHY). Statistical significance levels are represented as ⁣^∗^*p* < 0.05, ⁣^∗∗^*p* < 0.01, and ⁣^∗∗∗^*p* < 0.001.

Furthermore, we evaluated the efficacy of QXHY in preserving cellular interconnections in SAP mice regarding their structural integrity under TEM. As expected, the gaps between IECs significantly enlarged in the SAP group compared to that in the Sham + Veh group (shown in Figure 3b). In contrast, mice treated with QXHY showcased the maintenance of IEC junction integrity (shown in Figure 3c).

Subsequently, the expression of TJs, encompassing Claudin-1, Occludin, and ZO-1 was assessed using both qRT-PCR and Western blotting techniques. The results revealed a significant reduction in Claudin-1, Occludin, and ZO-1 mRNA expression levels within the SAP model mice (shown in Figure 3e). These changes were counteracted by QXHY treatment in SAP mice. In the subsequent phase of analysis, the expression of these protein components was scrutinized within the ileum tissue, revealing a substantial attenuation in their expression within the SAP group. On the other hand, a significant augmentation was observed in the QXHY-treated group (shown in Figure 3f). This trend was comparable with the qRT-PCR findings, hence underscoring the congruence between these investigative techniques.

### 3.4. QXHY Restores the Reduced Ileal ILC3s and Increases the Expression of IL-22 and IL-17 mRNA

Previous research confirmed that IL-22, derived from a diverse array of lymphocytic sources within the innate and adaptive immune systems, including effector CD4+ T lymphocytes, predominantly Th17 cells, and ILC3s, exerts regulatory command over antimicrobial peptide synthesis and fucosylation processes in epithelial cells.

**Figure 4:**
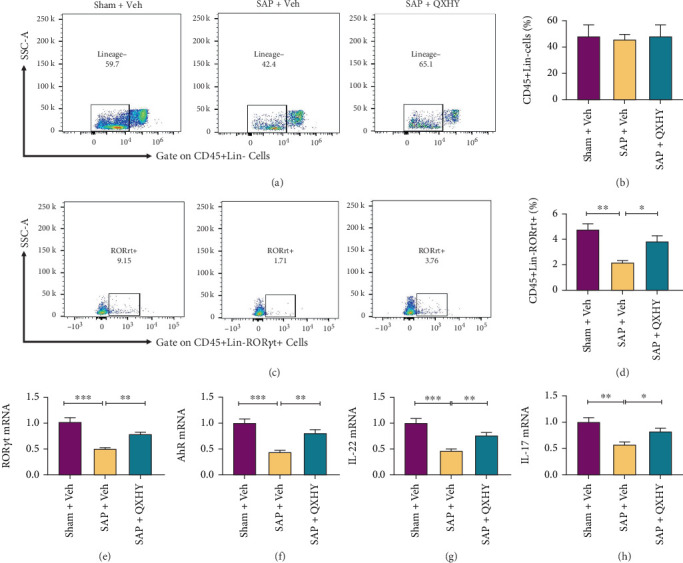
Restoration of ILC3 number and function by QXHY treatment in SAP mice. (a, b) Illustration of the gating strategy employed in the assessment of the ILC population in the small intestine and corresponding percentage. (c, d) Schematic display of the gating strategy utilized to analyze the ILC3 population in the small intestine and the respective percentage distribution. (e–h) Quantification of relative mRNA expression levels of ROR*γ*t, AhR, IL-22, and IL-17 as determined by qRT-PCR. Data are presented as either representative images or as the mean ± SD of distinct rat groups (*n* = 5 per group) from three independent experiments: Sham + Veh, sham surgery + Veh group (Sham + Veh); SAP + Veh, the SAP model + vehicle treatment group (SAP + Veh); and QXHY, the SAP + QXHY treatment group (SAP + QXHY). Statistical significance levels are represented as ⁣^∗^*p* < 0.05, ⁣^∗∗^*p* < 0.01, and ⁣^∗∗∗^*p* < 0.001.

**Figure 5:**
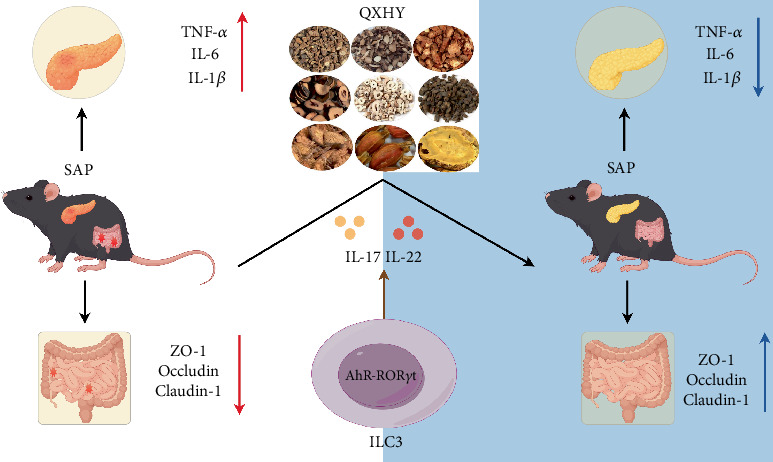
Schematic diagram of the possible mechanisms of QXHY to ameliorate SAP symptoms. The figure was drawn by Figdraw.

Through flow cytometry, our initial analysis involved the totality of ILC populations (CD45 + Lin- cells). Intriguingly, this analysis unveiled uniformity in total ILC representation across tripartite experimental cohorts (shown in Figure 4). In parallel, QXHY administration had no effect on ILC1s or ILC2s, distinct categories of ILCs (shown in Figure 4). Unlike the control, the SAP group had an incipient drop in tissue-resident ILC3 numbers (shown in Figure 4). Consistent with anticipation, intervention with QXHY caused a significant rejuvenation in the frequency and abundance of ROR*γ*t-ILC3s, effectively ameliorating their numbers (shown in Figure 4). Intriguingly, we assessed the two pivotal transcription factors, ROR*γ*t and aryl hydrocarbon receptor (AhR), intrinsic to the functioning of ILC3s. SAP imposition induced an inhibitory effect on ROR*γ*t and AhR mRNA expression within the ileal milieu, as shown by their attenuated levels relative to comparator cohorts. Conversely, the QXHY-treated group had an appreciable preservation of ROR*γ*t and AhR mRNA abundance (shown in Figure 4). We eventually focused on assessing IL-22 and IL-17 mRNA content within the ileal tissues. Under basal conditions, ILC3s were recognized for their basal IL-22 production, albeit with minimal IL-17 synthesis (shown in Figure 4). We noted a substantial attenuation in both IL-22 and IL-17 transcript levels within the ileal tissues of SAP-affected mice. Specifically, QXHY administration displayed the capacity to reverse the dwindling levels of IL-22 and IL-17 within the ileal tissue of SAP-affected mice. Collectively, these findings corroborate the regulatory effect of QXHY on the intestinal immune landscape, specifically in its adeptness to invigorate the ROR*γ*t+-ILC3 subpopulation in response to SAP-instigated intestinal damage (Figure 5).

## 4. Discussion

AP is often triggered by complex etiological factors involving different mechanisms and lacks successful treatment [32, 33]. Previous clinical observations in patients with SAP have reported associations between the use of QXHY and improvements in inflammatory markers and gastrointestinal function. However, the mechanisms underlying these potential effects are not fully understood. Growing evidence suggests that intestinal barrier dysfunction contributes to systemic organ injury in SAP. Consequently, therapeutic strategies aimed at preserving intestinal integrity have gained research interest. In this study, we investigated the effects of QXHY on intestinal barrier damage in a mouse model of SAP. Our results suggest that QXHY may attenuate SAP-induced intestinal injury, potentially through mechanisms involving the restoration of ILC3s.

As the primary defense against pathogens, the intestinal barrier relies fundamentally on the structural integrity of the intestinal epithelium, which consists of IECs and their intercellular TJs. A marked decrease in TJs expression is a key factor leading to increased intestinal permeability. This elevated permeability is widely recognized to facilitate the translocation of bacteria and ETs from the intestinal lumen into the mesenteric lymphatic system and subsequently into the systemic circulation, thereby exacerbating systemic inflammatory responses and potentially contributing to multiple organ dysfunction and failure [34]. These observations underscore a critical interaction between inflammatory mediators and intestinal barrier integrity, wherein inflammation significantly compromises barrier function. Thus, inhibiting inflammatory cascades represents a plausible therapeutic strategy to mitigate intestinal barrier injury. In the present study, we observed significant elevations in pancreatic MPO, serum AMY, and proinflammatory cytokines (TNF-*α*, IL-1*β*, and IL-6) in mice with SAP, which correlated with aggravated pancreatic and intestinal tissue damage. These findings confirm that the model successfully recapitulates key features of SAP-associated intestinal injury. Treatment with QXHY significantly reduced these key pathological indicators and alleviated tissue damage in both the pancreas and small intestine. These results support the potential utility of prophylactic intervention in reducing the incidence and severity of SAP. Furthermore, these pivotal findings motivate further investigation into the molecular mechanisms underlying the anti-inflammatory effects of QXHY.

Increased intestinal permeability is an early and prominent sign of initial impairment in intestinal barrier function, microscopically characterized by disruption of TJs. The perturbation of TJs during SAP has been extensively documented in animal studies [35, 36]. Clinical evidence also indicates decreased expression of key TJ proteins—including Claudin-1, Occludin, and ZO-1—in colonic mucosal tissues of SAP patients compared to healthy individuals [37, 38]. Furthermore, SAP patients with detectable BT exhibit significantly lower Occludin and ZO-1 levels than BT-negative patients [39], confirming a direct relationship between TJ disruption and BT in SAP. Therefore, alleviating TJ damage is essential for restoring intestinal barrier integrity in SAP.

In this study, we performed TEM to evaluate TJ morphology in SAP model mice. TEM revealed narrower intercellular spaces between IECs in QXHY-treated mice than in untreated SAP controls, indicating a protective effect of QXHY on TJ ultrastructure. Consistent with these morphological observations, RT-PCR and Western blot analyses showed that SAP mice exhibited downregulation of Claudin-1, Occludin, and ZO-1 at both transcriptional and protein levels compared to the Sham + Veh group. QXHY treatment significantly elevated the expression of all three TJ components, effectively counteracting SAP-induced suppression. In addition, reduced serum levels of lipopolysaccharide (LPS) and DAO in the QXHY group provided further evidence of its barrier-stabilizing role in SAP.

As a distinct population of innate immune cells, ILCs are crucial regulators of tissue homeostasis and repair. They are strategically localized at mucosal interfaces, including the intestinal and respiratory tracts, where their close association with epithelial cells enables a rapid response to environmental challenges. This interaction supports tissue regeneration and orchestrates early immune defense, mechanisms essential for the preservation and restoration of tissue equilibrium [40].

Among ILCs, ILC3s originate from fetal liver progenitor cells during embryogenesis and rapidly colonize barrier tissues, particularly the intestinal mucosa, where they constitute the dominant ILC subset and play a key role in maintaining intestinal homeostasis [41, 42]. Characterized by the expression of the transcription factor ROR*γ*t, ILC3s are activated via the AhR by stimuli such as IL-23 and IL-1*β*, which are influenced by microbiota-derived signals. Upon activation, ILC3s secrete IL-22 and IL-17. IL-22 in turn enhances barrier integrity by promoting the production of antimicrobial peptides (e.g., RegIII*β*, RegIII*γ*, and S100 proteins) and stimulating mucin secretion from goblet cells [43, 44]. Furthermore, IL-22 inhibits IEC apoptosis through mechanisms involving fucosylation modulation or regulation of antiapoptotic proteins including Bcl-2, Bcl-xL, and Mcl-1 [45, 46].

Furthermore, IL-22 enhances the synthesis of TJ proteins, thereby contributing to intestinal homeostasis. During the reparative phase of intestinal inflammation, ILC3s serve as a paramount source of IL-22; its deficiency can lead to microbial dysbiosis, barrier disruption, and increased susceptibility to colitis in mice. Similarly, IL-17—also produced by ILC3s—can influence the inflammatory immune response by activating secretory and growth factors in intestinal epithelial and endothelial cells and has been shown to independently modulate and protect the intestinal mucosal barrier via regulation of TJs [47–49]. The regenerative capacity of the intestinal epithelium, which is critical for barrier function, relies on the proliferation and differentiation of intestinal stem cells (ISCs). Notably, ISCs highly express IL-22R. Interaction between IL-22 (largely derived from ILC3s) and IL-22R promotes the proliferation and repair of LGR5+ ISCs, thereby facilitating the regeneration of damaged intestinal epithelium [50].

In the present study, QXHY treatment demonstrated a protective effect against SAP-induced TJ injury and barrier dysfunction. This was evidenced by mitigated pathological damage in the intestinal mucosa, reduced serum markers of intestinal injury (DAO, D-LA, and LPS), and upregulation of key TJ proteins (Claudin-1, Occludin, and ZO-1). Although the overall proportion of intestinal ILCs was not markedly altered in SAP mice, the specific subset of ILC3s and the levels of their associated cytokines (IL-22 and IL-17) were significantly decreased. Intriguingly, QXHY treatment increased the proportion of ILC3s and enhanced the levels of IL-17 and IL-22 in the intestinal mucosa. These results suggest that QXHY ameliorates SAP-induced intestinal barrier damage, potentially by modulating ILC3s and restoring their cytokine profile.

This study has certain limitations. We only tested a single dose of QXHY and evaluated it at one time point in this study. To better validate the therapeutic effects of QXHY, future experiments should incorporate multiple dosages and assess the QXHY-treated group at additional time points. Ideally, a market-available positive control drug would be included to compare the efficacy of QXHY. However, no specific medicine exists for treating pancreatitis, with conventional treatments for AP primarily aimed at alleviating symptoms and complications associated with AP. Using these treatments as controls in mouse models poses challenges. Furthermore, this study lacks the mechanistic study of QXHY, and the mechanism of action of QXHY needs to be studied in the future.

## 5. Conclusion

This investigation reveals novel insights into the molecular processes underlying the efficacy of herbal medicine. Our results show that SAP induced damage on epithelial cell TJs and disrupted intestinal barrier function in mice. However, QXHY treatment ameliorated this phenomenon, mainly by modulating the population and functional phenotypes of ILC3s in the intestinal tract in SAP-affected mice.

## Figures and Tables

**Table 1:** The primer sequences for qPCR.

**Genes**	**Primers**	**Length**
Mouse-claudin1-F	TGGGTTTCATCCTGGCTTCT	133
Mouse-claudin1-R	GACATCCACAGTCCCTCGTA	
Mouse-occludin-F	TGAAAGTCCACCTCCTTACAGA	116
Mouse-occludin-R	CCGGATAAAAAGAGTACGCTGG	
Mouse-ZO1-F	ACGATCTCCTGACCAACGTT	159
Mouse-ZO1-R	GCTTTGGGTGGATGATCGTC	
Mouse-ROR*γ*t-F	TCCCGAGATGCTGTCAAGTT	104
Mouse-ROR*γ*t-R	ACTTGTTCCTGTTGCTGCTG	
Mouse-AHR-F	GCCCTTCCCGCAAGATGTTAT	117
Mouse-AHR-R	TCAGCAGGGGTGGACTTTAAT	
Mouse-IL17-F	ACTCTCCACCGCAATGAAGA	161
Mouse-IL17-R	CTCTCAGGCTCCCTCTTCAG	
Mouse-IL22-F	ATGAGTTTTTCCCTTATGGGGAC	124
Mouse-IL22-R	GCTGGAAGTTGGACACCTCAA	
Mouse-GAPDH-F	AGGTCGGTGTGAACGGATTTG	123
Mouse-GAPDH-R	TGTAGACCATGTAGTTGAGGTCA	

## Data Availability

The data that support the findings in our study are available from the corresponding authors upon reasonable request.
